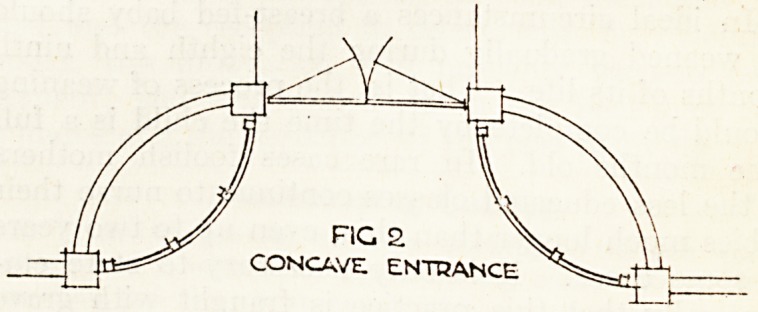# Sanatoria and Landscape Architecture

**Published:** 1912-10-19

**Authors:** 


### HOSPITAL ARCHITECTURE AND CONSTRUCTION.

[Communications on this subject should be marked "Architecture" in the left-hand top corner of the envelope.]

### Sanatoria and Landscape Architecture.

Landscape architecture as an art has no definite accepted standard; this may reasonably be accounted for by the varying popular ideas of what a good landscape should possess; the dweller in hilly or moorland country has fixed notions depending on his environment, and is at a loss to appreciate the beauties of flat-wooded country, and one accustomed to appreciate a view of the sea is similarly at a loss to appreciate the outlook which has no waterway. This diversity of taste gives opportunity to the landscape architect to plan grounds that will appeal to all lovers of Nature. Owing to recent legislation it may safely be forecasted that such skill will be in demand for the provision of establishments to accommodate 9,000 tuberculous patients in the immediate future; it would also seem possible that the exercise of these patients could be amenably reconciled to the manual labour required in laying out and planting the grounds of the several colonies to be formed.

The ideal site for an institution will have its boundaries of fairly regular lines forming an oblong figure, of which the greatest length is approximately north and south, and the length about one-third greater than its width. Along the north-east or north-west boundary a public road should give access to the buildings, for this aspect is of least consequence in regard to views, and the warmer and better aspects can be kept open and private. Shelter from injurious winds will possibly be afforded by a range of hills or belt of timber on the north-east and north-west boundaries. In selecting a site that which is already well wooded should be preferred rather than one which has no trees, as thinning out timber is an easy and economical process, if not even remunerative, whereas timber growing is both expensive and slow. The natural developments of an individual site must be considered, and this particularly as no two sites will offer exactly the same problem; this consideration will include the opening up of prospects, aspect, contours, landmarks, and waterways; all these factors play some part in determining the line of the main approach. It should have an ascending gradient, particularly as the buildings are neared, and it may be described as bearing the same relation to the grounds that the entrance hall does to the buildings, for from it access should be possible to all outlying and detached offices, such as the mortuary or laundry blocks. Though it must fufil this condition it must without equivocation be apparently the shortest route from the public highway to the main portico, therefore if its line be circuitous in order to obtain an easier gradient the fact should be masked by some apparently natural obstacle; if this is not attended to an unrecognised, but more direct, path will soon be trodden by those who frequently traverse the approach. Care should be taken to subordinate minor drives and walks leading from the main approach to outlying buildings so that strangers will not be tempted to follow them; this may conveniently be done by tending them away sharply soon after they leave the main avenue. As a rule the entrance gateway is placed at right angles to the high road in order to gain dignity, though the drive may turn in another direction very soon to ensure privacy. The entrance boundary walls or fences are usually convex in plan, as this is the natural line of approach (see fig. 1). This may, however, be varied and often bettered by a concave form of boundary, and a post and chain railing to maintain the convex line of drive, with lawn or shrubbery as a filling (see fig. 2).

It will sometimes be desired to combine with the entrance gates a lodge, and if this is done both the high road and the drive should be clearly observable from the lodge windows, so that delay in attending the gates is not occasioned.

The gradient of a drive or approach ought not to exceed one in fourteen, and it is useful to remember

FIG 1

CONVEX APPROACH

FIG 2

CONCAVE ENTRANCE

that nine feet is the minimum width for a single carriageway, and that two carriages can pass in fourteen feet. The gravelled plane or terrace in front of the entrance doors should not be less than thirty-three feet.

The drive had better not skirt the boundaries more than necessary, as this tends to emphasise want of extent and variety; but as economy will enter largely into sanatoria problems it is not likely that expensive approaches will be made longer than is absolutely necessary.

Views of rich distances can be a feature of the drive, and particularly when a site is well wooded this is economically effected by cutting down a few trees, and the view of the landscape as the ground falls towards the south should amply repay the small outlay. In conclusion, it may be said that the features of the approach, as of all other items in the grounds, should be boldly planned, for Nature is always at work modifying and toning down man's handiwork.

## Figures and Tables

**Fig 1 f1:**
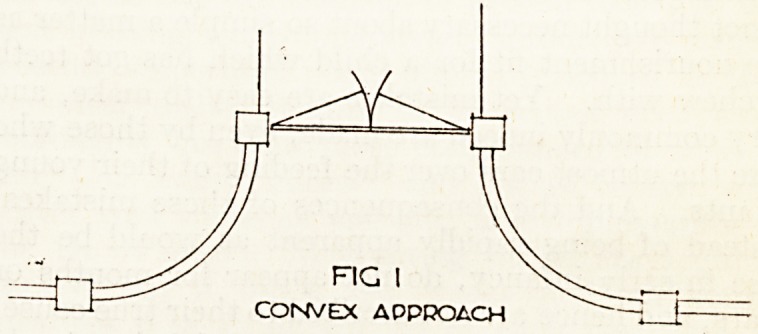


**Fig 2 f2:**